# Can a GP be a generalist and a specialist? Stakeholders views on a respiratory General Practitioner with a special interest service in the UK

**DOI:** 10.1186/1472-6963-6-62

**Published:** 2006-05-30

**Authors:** Mandy A Moffat, Aziz Sheikh, David Price, Annie Peel, Siân Williams, Jen Cleland, Hilary Pinnock

**Affiliations:** 1 Department of General Practice and Primary Care, University of Aberdeen, Foresterhill Health Centre, Westburn Road, Aberdeen AB41 6ST, UK; 2 Division of Community Health Sciences: GP Section, University of Edinburgh, 20 West Richmond Street, Edinburgh EH8 9DX, UK; 3 De Montfort University, Faculty of Health & Life Sciences, Hawthorn Building 0.15b, The Gateway, Leicester, LE1 9BH, UK; 4 30 Uplands Road, London N8 9NL, UK

## Abstract

**Background:**

Primary care practitioners have a potentially important role in the delivery of specialist care for people with long-term respiratory diseases. Within the UK the development of a General Practitioner with Special Interests (GPwSI) service delivered within Primary Care Trusts (PCTs) involves a process of 'transitional change' which impacts on the professional roles of clinicians who may embrace or resist change. In addition, the perspective of patients on the new roles is important. The objective of the current study is to explore the attitudes and views of stakeholders to the provision of a respiratory GPwSI service within the six PCTs in Leicester, UK.

**Methods:**

Using a qualitative design, GPs, nurses, secondary care doctors, nurse specialists, physiotherapists, a healthcare manager and patients with respiratory disease took part in focus groups and in-depth interviews.

**Results:**

The 25 participants expressed diverse opinions about the challenge of integrating specialist services with generalist care and the specific contribution that GPs might make to the care of people with chronic respiratory disease. A range of potential roles for a respiratory GPwSI, working as part of a multi-disciplinary team, were suggested, and a number of practical issues were highlighted. Success of the GPwSI role is deemed to be dependant on having the trust of their primary and secondary care colleagues as well as patients, credibility as a practitioner, and being politically astute thereby enabling them to act as a champion supporting the transition process within the local health service.

**Conclusion:**

The introduction of a respiratory GPwSI service represents a challenge to traditional roles which, whilst broadly acceptable, raised a number of important issues for the stakeholders in our study. These perspectives need to be taken into account if workforce change is to be successfully negotiated and implemented.

## Background

The NHS Plan signalled the creation of General Practitioners with Special Interests (GPwSI) in the UK.[1] A GPwSI was initially conceived as a GP who supplemented their important generalist role by delivering services beyond the scope of traditional primary care, with the primary aim of reducing waiting lists for specialist opinions.[2] The importance of maintaining a primary care perspective while enhancing specialist competencies is an important theme of early conceptual work.[2,3]

Globally, healthcare services are reconfiguring to meet the increasing challenge of providing care for people with long-term disease.[4,5] Predicted to become a leading cause of morbidity and mortality by 2020, [6] respiratory disease already represents a considerable burden on hospitals, especially in winter.[7,8] New models of providing intermediate care, often involving enhanced nursing services are already being developed.[9,10] The potential role of a respiratory GPwSI has been described, [11-13] and previous work has shown that the Primary Care Trusts (PCTs) – statutory bodies who commission care for populations of approximately 100,000 in the UK – are increasingly considering a respiratory GPwSI service as a means of reducing pressure on secondary care services and to raise standards in primary care.[14] A recent survey of referrals to a secondary care respiratory clinic concluded that about a quarter of patients could potentially be seen by a respiratory GPwSI, providing support for this approach.[15]

**Figure 1:**
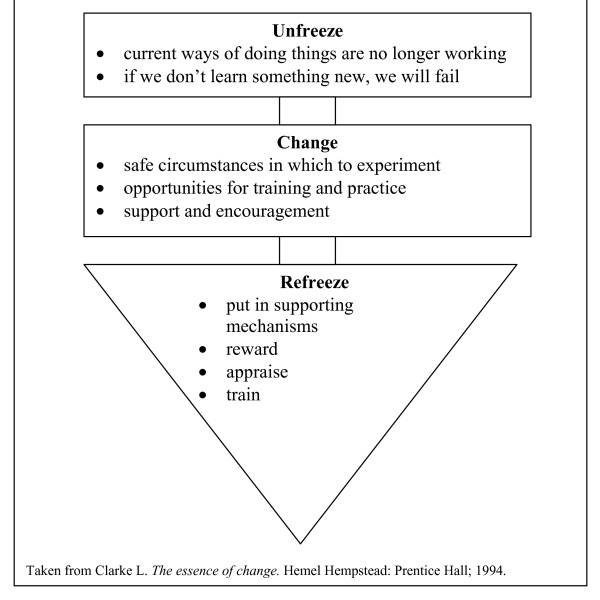
The unfreeze-change-refreeze model.

The development of a GPwSI service involves a process of 'transitional change' as a new organisational structure is implemented over a controlled period of time.[16] The "unfreeze-change-refreeze" model originally defined by Lewin, [17] but further explored by other authors sums up the complex issues underlying transitional change within organisations and subsequent success or failure (see Figure 1).[18] Successful facilitation of change involves an understanding of the change process which Clarke describes in terms of 'structure', 'process' and 'people'. The importance of 'people', both as a source of inertia and a source of leverage for change, is highlighted by many recognised models of change, and may be particularly significant in the context of healthcare services as influential professional groups embrace or resist change.[16] For example, the GPwSI role may be resented as a threat to traditional specialist roles (from within primary and secondary care) or welcomed as additional resource, perceived as undermining the specialism of general practice or embraced as a opportunity for professional development.[3] It has been suggested that GPwSIs may contribute clinical leadership to this development agenda.[19]

This qualitative study is part of a collaborative programme of work exploring the role, service delivery implications and training needs of respiratory GPwSIs. It complements a recent national survey which reported that although only six percent of PCTs already had a respiratory GPwSI in post, a further 32% indicated that they were considering developing a respiratory GPwSI service.[14] Current NHS policy emphasises the importance of including the patients' perspective on planned service development.[20] It is therefore timely to seek to understand the views of all relevant stakeholders, to inform the process of change and enable the implementation of a new service that will be acceptable to patients and to healthcare professionals.

The aim of the current project was to explore attitudes and views of primary and secondary care clinicians, health service managers and patients with respiratory disorders to the provision of a respiratory GPwSI service in their local area.

## Methods

Our study was undertaken in 2005, in Leicestershire, UK, with the approval of Grampian and Leicester research ethics committees. Research governance approval was granted by the Leicester Primary and Acute Care Trusts.

### Setting

Leicestershire includes six PCTs serving a population of about a million people covering both city and rural environments and representing populations with contrasting demographic features including areas of deprivation/affluence and minority ethnic groups. The rates for respiratory disease are average for the UK, apart from a higher incidence of tuberculosis [Scullion J. Personal Communication, 2004]. At least one PCT was known to be considering the appointment of a respiratory GPwSI.

### Design

We used a qualitative research design employing a range of data collection methods in order to encourage participation and to include a wide spectrum of views. Focus groups were used initially, these supplemented by face-to-face and telephone interviews to target those groups that were felt to be under-represented.[21] A short report detailing themes was then e-mailed for feedback to all GP practices in one PCT area.[22,23]

We purposively sampled participants to represent a range of jobs, roles, grades, geographical/demographic areas, and known attitudes towards GPwSIs (through identification from other participants). Those approached and those subsequently took part can be seen in Table 1.

The focus groups and interviews were semi-structured in nature. Based on our previous work, [13,14] and our understanding of the literature, we devised questions to stimulate discussion on the perceived roles, benefits, concerns, outcomes and evaluation of a possible respiratory GPwSI service (see Appendix 1 for an example topic guide). The 'GPwSIs initiative' was briefly explained to those participants not already familiar with the concept

Focus groups and interviews were recorded with the consent of the participants, transcribed and transcripts checked for quality by the main researcher (MM) before being entered on N-Vivo. Initial themes were identified from the focus groups and were used to inform subsequent interviews. N-Vivo was then used to aid data management and coding using an agreed framework. Excel was used to construct an overall matrix to allow cross-case and within case comparisons.[24,25] Analysis was carried out by the main researcher (MM) with discussion regarding emerging frameworks and coding strategies taking place at regular intervals with other members of the team (HP, JC, AP). Disagreements were resolved through discussion.

## Results

Twenty five stakeholders in total took part, encompassing patients with asthma and/or Chronic Obstructive Pulmonary Disease (COPD) (n = 7), secondary care nurses and allied health professionals (n = 6) primary care nurses (n = 4), general practitioners (n = 3), secondary care doctors (n = 3), a PCT manager and a non-Leicester respiratory GPwSI. One of the Leicester GPs was also a GPwSI (non-respiratory). Four focus groups, three face-to-face interviews and four telephone interviews were used to collect data (see Table 1). The key themes were fed back by e-mail to all the GP practices (n = 28) in one of the PCTs: no dissenting responses were received.

### Can a generalist be a specialist?

There was some ambivalence within the different focus groups and interviews as to whether a GP could also be a specialist. Even the title 'GP with a Special Interest' was challenged.

*"You'd have to do away with the title GP for a start. If you're talking about a specialist then he's a specialist" *(Patient)

*"The word 'specialist interest' will disappear, because it is not the right word, just because you have got an interest, but some of them they are going to be a specialist in time, the interest will vanish and they become a specialist in that arena" *(GP)

At a theoretical level, it was suggested by some participants that generalist skills could usefully broaden the perspective of specialist consultations, especially in patients with co-morbidity.

*".they [GPs] understand where the patient is going through all sorts .., how their heart failure is getting on, how their diabetes is being managed, and take the patients who may have 3 or 4 illnesses. We don't often see the whole picture purely because we can't." *(Respiratory physician)

*"... if everybody specialises ... you would go to the diabetic GP, the heart GP, the respiratory GP, and who is left to know the patient as a whole?" *(Primary care nurse)

Some nurses, however, disputed the holistic role of GPs and thought that this should be the role of nurses, though nurse specialisation (e.g. in the UK, nurses are increasingly providing chronic disease management in both primary and secondary care [24]) was seen as a threat to that important role.

*"Maybe that's quite a key point actually isn't it, that a GP because of their training is probably not capable of looking at them holistically and with chronic disease such as this they need holistic care to keep them out of that revolving door syndrome" *(Secondary care nurse)

*"And it maybe that the nurses are asking to do that, but a lot of nurses are specialised now and do respiratory and all that*..." (Primary care nurse)

Both patients and professionals expressed concern about the potential adverse effect specialism might have both on the individual's generalist skills and on the future of general practice.

*"If Dr [GP], we'll say for arguments sake he was an, if he was upgraded to a specialist in asthma or breathing problems wouldn't that then diminish the GP side of this practice?" *(Patient)

*"deskilling from a GP" *(Secondary care allied health professional)

*"GP's will have portfolios in the future, I do not, I cannot foresee a generic GP, as we know them, carrying on delivering the NHS Plan" *(GP)

### The roles of a GPwSI

A variety of possible roles were suggested as being appropriate for a GPwSI to deliver. For example:

• Providing a clinical service for patients, that would be "*local, and easily accessible" *with a familiar doctor in whom patients felt they could *"have more confidence"*

• Providing education and support, as a clinician who was "*respected of his colleagues, and would oversee and would be approachable and helpful, a trainer and co-ordinator." *'Bridging the gap' between primary and secondary care was specifically highlighted: *"to set, you know minimum standards of what GP's can and can't manage..., and to also say to the hospital doctors, 'look this is what we would like you to do with these patients."*

• Co-ordinating services: *"a specialist team, working in the community"*

• Developing new services: e.g. pulmonary rehabilitation, intermediate care. a point of referral midway between primary and secondary care

Opinions were divided on the potential contribution GPs could make to specialist care. One healthcare professional felt that a respiratory GPwSI role would not be as large as in other clinical areas because it was well supported by guidelines and was either very routine (and therefore easily delegated to nurses) or very complicated (and therefore should be in the hospital). By contrast, others identified a 'referral hierarchy' which could include GPwSIs.

*"Medicine has become [a] very protocol [driven], and what you actually need is someone who can follow the guidelines accurately and occasionally think outside that particular box you know without having to refer up, I am confident that GP's can do that." *(Respiratory physician)

*"I would sooner see someone that knows a little bit more because [GP] doesn't specialise in asthma. She refers you to [Practice Nurse] but I, you know, I mean very, I think [PN]'s wonderful as well, but if I was having more problems then perhaps I would like to see someone that a doctor that, you know, is just that little bit more advanced than what the nurse is" *(Patient)

*"Well I still see the very severe and complicated ones coming in to the secondary care, whereas the bulk of the, that middle stage would go to a specialist GP, or a specialist clinic, that would have specialist nurses..." *(Primary care nurse)

The local needs of the area were seen as paramount, with general agreement that the role should be clear and avoid duplication of already existing services. Secondary care participants thought that although gaps were still evident, in comparison to some areas of the UK, Leicester was fortunate to have relatively few gaps.

The effect on workload was debated both by patients, who were concerned that their GP was already too busy, and professionals who raised concerns that offering a GPwSI service might not just meet existing need but actually create demand.

*"There's certainly a role but would he be able to cope with his normal influx of patients who keep him busy all week now anyway..." *(Patient)

*"The workload might increase, because if the patients realise that there is more contact available, they might ask for this contact." *(Respiratory physician)

### The right person for the job?

GPs (or medical doctors generally) were thought to have more power compared to other healthcare professionals when it came to influence over and education of other doctors as well as being able to influence decisions at PCT level. Doctors' ability to prescribe was also seen as an important and powerful lever. This was highlighted several times in the context of comparing the roles of specialist nurses versus GPwSI. For example:

*"...if they are going to be a champion, politically, the more clout they carry, the better really" *(Secondary care allied health professional)

*"...you need a doctor's voice as well.....that is why a lot of initiatives have failed in primary care because there hadn't been that ground swell from within. You can talk until you are blue in the face as a nurse....<snip> sometimes you need to have that label" *(Primary care nurse)

*"it could be a GP not convinced it has to be a GP...<snip> need to make sure [whoever does it] has the appropriate training, backing and empowerment to provide that service and not have to stand in the corridor saying can you sign this prescription for beclomethasone or whatever." *(GP)

Throughout the interviews and focus groups the fact was highlighted that whoever takes on the role of a GPwSI needs to have the trust of their primary and secondary care colleagues as well as the patients, if the role is to succeed. Some of the personal qualities thought to be important for a GPwSI can be seen in Text Box 1.

### Making it work: possible issues and pitfalls

Highlighted in the majority of focus groups and interviews is that a GPwSI service should be a multidisciplinary team effort. A GPwSI working on their own was thought unlikely to succeed e.g. *"extremely difficult for any one person these days to have all the knowledge" *(GP). As well as teamwork *("the GPwSI cannot work without a team of nurses with them" *-GP), good links, support and communication with secondary care, primary care and PCTs was seen as pivotal with regards to the success of a GPwSI service.

Taking the wider context into account, another participant pointed out that what was needed was a:

*"...general buy-in from the whole health community, for that particular initiative, so that they become part of the wider team of professionals, in helping to tackle the problem of respiratory disease." (*PCT manager)

Several stakeholders suggested that emergency cover out of normal clinic hours would be a positive addition to the role of a respiratory GPwSI respiratory care. However, in the light of the new General Medical Services contract in the UK which has allowed GPs to 'opt out' of providing out-of hours care, views were mixed as to whether this would acceptable to potential GPwSIs:

*"Well, the role that they could play is there is enough of them to be on call, it is a hospital admission avoidance, and particularly if they are in areas where there is intermediate beds." *(GP)

*"it [GPwSI service] would probably need to be 24 hrs, and all the other bits that go with it" *(Primary care nurse)

*"if you are going to take some of that responsibility away from secondary care, em, I don't think it would hurt [being on call], but obviously the GP culture now..." *(Respiratory physician)

Another key point, made by both patients and professionals, was the need for a respiratory GPwSI to have access to appropriate equipment and facilities (e.g. radiology, lung function testing) to enable them to undertake a specialist role.

## Discussion

The introduction of a respiratory GPwSI service represents a challenge to traditional roles which, whilst broadly acceptable, raised a number of important issues for the stakeholders in our study. The challenge of integrating specialist services with generalist care and the specific contribution that GPs (as opposed to other healthcare professionals) might make to the care of people with chronic respiratory disease stimulated diverse opinions, as well as highlighting a range of practical issues. These perspectives will need to be taken into account if workforce change is to be implemented successfully.

### Limitations of the study

Our study was undertaken within one county of the UK, and our findings may not be wholly representative of attitudes in other areas where existing respiratory services may be more, or less, developed. However, Leicestershire offers areas with contrasting demography and a generally average profile of respiratory disease.

Early delays due to the recently introduced research governance regulations restricted the choice of times and venues that we were able to offer for the focus groups. However, by providing flexible alternatives (e.g. telephone or face-to-face interviews) we enabled the participation of a broad range of professionals. Although PCT managers are under-represented, two of the GPs additionally held managerial roles in their PCTs and therefore contributed to the management perspective.

We were aware that the positive attitude of the researchers to the GPwSI initiative might bias our interpretation and specifically aimed to foster an environment that allowed participants to air both negative and positive attitudes towards a respiratory GPwSI service.

### Strengths of the study

The main strength of the study is the breadth of participants, including patients, some of whom had experience of a GP 'champion' (although not officially a GPwSI), primary and secondary care clinicians and PCT management. By focusing in one area we were able to gain multiple perspectives on the potential contribution of a respiratory GPwSI on local services. Leicestershire did not have a respiratory GPwSI in post at the time, so that specific personality issues could not have affected the perceptions of our respondents. Interviews with a Leicestershire GP working as a GPwSI in another speciality and a respiratory GPwSI from another area with similar demography ensured that the perspectives of the GPwSIs were included.

The research team, including GPs, non-clinicians, social scientists with expertise in qualitative research, and people with experience of working within health service management and a UK respiratory charity, all contributed to the interpretation of the data ensuring a multi-disciplinary approach.

### Interpretation of findings in relation to previously published work

Our participants struggled to reconcile the generalist and specialist roles with concerns that ranged from the semantic (what do you call a 'general' practitioner who specialises?) to the practical (who would cover the GPwSI's existing general practice workload?). These issues echo the conceptual discussions led by the Royal College of General Practitioners who rejected the title 'GPs Specialists' on the grounds that all GPs are specialists in the 'generalist tradition of primary care'.[2] The principle that a GPwSI should be a GP first, and then a specialist underpins all the early guidance.[2] Our findings focus attention on a number of themes related to this concept.

Patients with long-term conditions often have co-morbidity[26] and GPs knowledge of their patients' medical and social background was cited with some envy from the perspective of secondary care. From their perspective, patients expressed confidence in seeing a GPwSI whom they knew and who, by implication, knew them and might be expected to be familiar with their medical/social needs. However, whilst some participants acknowledged the contribution general practitioner skills could bring to specialist consultations, others felt that the nurses were the more appropriate professionals to provide the holistic care needed. Interestingly, the recent trend to nurse specialisation was identified as a threat to that holistic role – a warning, perhaps, to GPwSIs who value their generalist skills in managing patients with co-morbidity. Increasing specialisation may even be construed as a threat to the whole concept of a 'generalist practitioner', [3] an opinion echoed by one of our participants.

The potential impact of the GPwSI initiative on the workload of general practice is highlighted in the earliest discussion documents, [2] and remains an important concern.[27] In our study the concern was raised most strongly by the patients who could foresee increasing difficulty making appointments with their chosen GP. A third of PCTs in a recent national survey identified lack of an 'available GP' as a barrier to implementing a respiratory GPwSI service, [14] making it one of the most significant obstacles that will need to be overcome if GPwSI services are to be developed.

The original role of GPwSIs, as conceived in the NHS Plan, was that 'specialist GPs' should accept referrals and undertake specific out-patient procedures.[1] Both patients and clinicians described a 'referral hierarchy' with generalist clinicians seeking the advice of specialist nurses, whilst more complex problems are referred to hospital specialists. Opinions were divided about where GPwSIs might fit into this hierarchy. In line with a recent study who concluded that about a quarter of referrals to respiratory consultants could be seen by a GPwSI, [15] many of our participants considered that a GPwSI could usefully provide advice on less complex issues. Others questioned whether they could contribute more than the existing specialist nurses. The ability of GPs to prescribe was a key factor, though changes in legislation allowing extended nurse prescribing and supplementary prescribing are blurring this distinction.[28] The need for a GPwSI to work within a multidisciplinary team, complimenting rather than duplicating secondary care and/or specialist nurse services, was highlighted by our participants. Further research should focus on the relative merits of these models of care.

An unexpected role raised by a number of participants, was that a GPwSI service should include an emergency and/or out-of-hours, element. One specific idea was the provision of in-patient care using a local community hospital in order to reduce the recognised pressure on acute hospital beds. There is growing evidence in support of 'Hospital at Home' schemes, normally led by specialist nurses, which can safely reduce admissions for acute exacerbations of chronic obstructive pulmonary disease in about a quarter of presentations.[29] A GPwSI service offering low cost intermediate care beds could potentially accept those patients currently admitted for predominantly social reasons. In the UK, under the terms of the new General Medical Services contract, PCTs have the responsibility of providing out-of-hours care[30] and could consider the potential of a GPwSI led service.

Many of the concerns expressed by our participants reflect the anxiety engendered by the 'unfreeze' phase of transitional change, as professionals are forced to question the value of their traditional service in the light of an evolving context, identify those components that will contribute to the new service (and by implication discard those that do not), and develop new skills that can enhance their role. Respected role models may facilitate this process.[16,18,31]

A common feature of many models of change is the emphasis on 'people' both as driving forces and restraining forces. Clarke highlights the importance of communication and 'getting everyone on board' during the process of transition.[18] GPwSIs, identified by a number of stakeholders in our study as a credible practitioner who can engage with both primary and secondary care, clinicians and managers, may have the potential to help 'unfreeze' the current set up, and enable change by providing trusted leadership during the 'transition phase'. This reflects early conceptual work which outlined a strategic role for GPwSIs, who were seen as having the potential to influence both clinical and managerial colleagues.[13] This process of workforce reconfiguration is the subject of an on-going study within PCTs in the UK.[32]

## Conclusion

The development of respiratory GPwSI services, currently being implemented or considered by a third of PCTs in the UK, involves a substantial process of change as professional roles adapt to the new models of care. A pre-requisite of successful transition involves understanding the issues from the perspective of all the stakeholders. Our findings suggest that whilst there are concerns that the development of specialist roles may compromise valued generalist skills, respiratory GPwSI have the potential to act as credible, local champions to support the transition process.

## Abbreviations

GP General Practitioner

PN Practice Nurse

GPwSI General Practitioner(s) with a Special Interest.

PCT Primary Care Trust. These organisations commission local healthcare services in England for populations of about 100,000.

NHS National Health Service

UK United Kingdom

<snip> Text in the transcription has been taken out to help make a point clearly but without losing meaning

## Competing interests

The author(s) declare that they have no competing interests.

## Authors' contributions

HP initiated the idea for the study and led the development of the protocol, and contributed to data analysis, interpretation of results and writing of the paper. MM was responsible for day-to-day management of the study, recruited the participants, data analysis and interpretation and wrote the first draft of the manuscript. AS, DP, SW, AP and JC contributed to the development of the protocol and the interpretation of results. All authors reviewed the final manuscript.

## Funding

Funded by a grant from the General Practice Airways Group (GPIAG).

## Pre-publication history

The pre-publication history for this paper can be accessed here:



## References

[B1] Department of Health (2000). The NHS Plan: a plan for investment, a plan for reform.

[B2] Department of Health and Royal College of General Practitioners (2002). Implementing a scheme for General Practitioners with Special Interests London.

[B3] Gerada C, Wright N, Keen J (2002). The general practitioner with a special interest: new opportunities or the end of the generalist practitioner?. Br J Gen Pract.

[B4] World Health Organisation (2002). Innovative care for chronic conditions: building blocks for action Geneva.

[B5] Improving chronic disease management. http://www.dh.gov.uk/assetRoot/04/07/52/13/04075213.pdf.

[B6] Murray CJ, Lopez AD (1997). Alternative projections of mortality and disability by cause 1990–2020: Global burden of disease study. Lancet.

[B7] British Thoracic Society (2001). The burden of lung disease.

[B8] Damiani M, Dixon J (2002). Managing the pressure: Emergency hospital admissions in London 1997–2001.

[B9] Department of Health (2005). Supporting People with Long Term Conditions: liberating the talents of nurses who care for people with long term conditions. Department of Health.

[B10] Taylor SJC, Candy S, Bryar RM, Ramsay J, Vrijhoef HJM, Esmond G, Wedzicha JA, Griffiths C (2005). Effectiveness of innovations in nurse-led chronic disease management for patients with chronic obstructive pulmonary disease: systematic review of evidence. BMJ.

[B11] Guidelines for the appointment of General Practitioners with a Special Interest in the delivery of clinical services: Respiratory Medicine. http://www.dh.gov.uk/assetRoot/04/06/83/77/04068377.pdf.

[B12] A working party of the GPIAG and RCGP (2003). General practitioners with a special interest in respiratory medicine. Prim Care Resp J.

[B13] Williams S, Ryan D, Price D, Langley C, Fletcher M, Everden P (2002). General practitioners with a special clinical interest: a model for improving respiratory disease management. Br J Gen Pract.

[B14] Pinnock H, Netuveli G, Price D, Sheikh A (2005). General Practitioners with a Special Interest in respiratory medicine: national survey of primary care organisations. BMC Health Services Research.

[B15] Gilbert R, Franks G, Watkin S The proportion of general practitioner referrals to a hospital respiratory medicine clinic suitable to be seen in a GPwSI respiratory clinic. Prim Care Resp J.

[B16] Iles V, Sutherland K (2001). Organisational change. A review for health care managers, professionals and researchers.

[B17] Lewin K (1952). Frontiers in group dynamics. Field theory in social science: Selected theoretical papers.

[B18] Clarke L (1994). The essence of change.

[B19] (2004). Royal College of Physicians of London, Royal College of General Practitioners, NHS Alliance. Clinicians, services and commissioning in chronic disease management in the NHS: the need for co-ordinated management programmes.

[B20] Department of Health (2005). Creating a Patient-led NHS – Delivering the NHS Improvement Plan. http://www.dh.gov.uk/assetRoot/04/10/67/40/04106740.pdf.

[B21] Curtis S, Gesler W, Smith G, Washburn S (2000). Approaches to sampling and case selection in qualitative research: examples in the geography of health. Soc Sci Med.

[B22] Silverman D (2000). Doing qualitative research: A practical handbook.

[B23] Kitzinger J, Pope C, Mays N (1999). Focus groups with users and providers of health care. Qualitative research in health care.

[B24] (2002). The Qualitative Research Unit, National Centre for Social Research. The conduct of qualitative depth interviews, publisher city?.

[B25] Miles MB, Huberman AM (1994). Qualitative data analysis: An expanded sourcebook.

[B26] Wright N, Smeeth L, Heath I (2003). Moving beyond single and dual diagnosis in general practice. BMJ.

[B27] Nocon A, Leese B (2004). The role of UK general practitioners with special interests: implications for policy and service delivery. Br J Gen Pract.

[B28] (2005). NHS Modernisation Agency and the Department of Health. Medicines Matters: A guide to current mechanisms for the prescribing, supply and administration of medicines. Department of Health.

[B29] Ram FSF, Wedzicha JA, Wright J, Greenstone M (2004). Hospital at home for patients with acute exacerbations of chronic obstructive pulmonary disease: systematic review of evidence. BMJ.

[B30] (2003). NHS Confederation, British Medical Association. New GMS contract 2003: investing in general practice.

[B31] General Practice in Airways Group (GPIAG) GPwSI Resource Pack: Learning from the experience of GPs and other practitioners with a special interest in respiratory medicine. http://www.gpiag.org/gpwsi/resource_pack_final_approved_version.pdf.

[B32] NHS Service Delivery and Organisation The process of planning, development and Implementation of a general practitioner with a special interest service in primary care organisations in England and Wales: a comparative prospective case study. http://www.sdo.lshtm.ac.uk/workforce.htm#specialinterest.

